# Topical AuNPs-Cys-Sm29 gel modulates the course of lesion development in experimental cutaneous leishmaniasis

**DOI:** 10.1371/journal.pntd.0013510

**Published:** 2025-09-22

**Authors:** Sayonara de M. Viana, Luciana Cardoso, Pedro B. Borba, Lucas P. Carvalho, Juvana Moreira, Fabio Mambelli, Sergio C. Oliveira, Edgar M. Carvalho, Camila I. de Oliveira

**Affiliations:** 1 Instituto Gonçalo Moniz, FIOCRUZ, Salvador, Brazil; 2 Departamento de Análises Clínicas e Toxicológicas, Faculdade de Farmácia, UFBA, Salvador, Brazil; 3 Instituto Nacional de Ciência e Tecnologia em Doenças Tropicais (INCT-DT), Salvador, Brazil; 4 Departamento de Biointeração, Instituto de Ciências da Saúde, UFBA, Salvador, Brazil; 5 Departamento de Bioquímica e Imunologia, Instituto de Ciências Biológicas, UFMG, Belo Horizonte, Brazil; 6 Departamento de Imunologia, Instituto de Ciências Biomédicas, USP, São Paulo, Brazil; University of Sao Paulo: Universidade de Sao Paulo, BRAZIL

## Abstract

The Sm29 antigen from *Schistosoma mansoni* has been shown to downregulate excessive inflammation associated with immune-mediated diseases. In contrast, cutaneous leishmaniasis (CL) caused by *Leishmania (Viannia) braziliensis* is marked by an inflammatory response that, when uncontrolled, contributes to disease pathology. In this study, we evaluated the therapeutic potential of topical rSm29 in combination with meglumine antimoniate (Sb^v^) in experimental murine CL. First, rSm29 was functionalized onto spherical gold nanoparticles using a cysteamine linker (AuNPs-Cys-*Sm*29). Topical application of this formulation of rSm29 significantly decreased ear lesion thickness, and the combination of topical AuNPs-Cys-*Sm*29 plus intraperitoneal Sb^v^ also significantly reduced ear lesion thickness, parasite load in the infection site, and the local inflammatory infiltrate when compared to mice treated with Sb^v^ only. The production of IFN-γ, TNF, and IL-10 was reduced in the draining lymph node, as well as the total number of CD3^+^CD4^+^IFN^+^ and CD3^+^CD4^+^TNF^+^ T cells in the infection site. This study demonstrated that combination therapy with topical AuNPs-Cys-*Sm*29 + systemic Sb^v^ reduced inflammation without compromising parasite clearance. These findings highlight the potential of AuNPs-Cys-*Sm*29 as a host-directed strategy in treating cutaneous leishmaniasis (CL).

## Author summary

In this work, we investigated a new treatment approach for a skin disease called cutaneous leishmaniasis (CL), caused by a parasite named *Leishmania braziliensis*, which triggers excessive inflammation and tissue damage. In this new treatment, we explored using a protein called Sm29, derived from another parasite, *Schistosoma mansoni*, known for its ability to reduce inflammation. We produced a cream consisting of AuNPs-Cys-*Sm*29 and used this cream to treat CL lesions, either alone or combined with the standard chemotherapy for CL (Sb^v^). The results showed that the combination therapy (AuNPs-Cys-*Sm*29 + Sb^v^) significantly reduced the severity of lesions, parasite numbers, and inflammation in the infected area compared to chemotherapy alone. We highlight that this anti-inflammatory effect did not interfere with the body’s ability to eliminate the parasites. These findings suggest that the Sm29 cream could be a promising addition to current CL treatments, offering a more efficient way for the patient to fight the disease.

## Introduction

Leishmania parasites transmitted by sand flies cause cutaneous leishmaniasis (CL); the disease has a variety of clinical presentations ranging from self-healing lesions to chronic disfiguring mucosal disease [1]. In Brazil, CL is caused mainly by *L. braziliensis* and is marked by an overt inflammatory response at the cutaneous site of infection, manifested by chronic lesions in which parasites may be scarce [2]. IFN-γ and TNF lead to macrophage activation and parasite control [3]; however, a poorly regulated Th1 response is associated with pathology. Importantly, chemotherapy with Meglumine Antimoniate (Sb^v^), the first line of drugs prescribed to treat CL in Brazil, is highly ineffective, and cure rates can be as low as 50% [4–6]. Therefore, targeting the host’s immune response has become increasingly investigated in CL caused by *L. braziliensis*, particularly in combination treatments, aiming to control the inflammatory response while providing anti-leishmania treatment [7].

Helminth infections and their products modulate Th2-immune responses associated with the pathology of allergic diseases [8]. Asthmatic patients infected with *Schistosoma mansoni* experience inhibition of the Th2 inflammatory response and a less severe course of disease [9]. This finding has been recapitulated experimentally, whereby *S. mansoni* antigens reduced the allergic Th2 response in a murine model of ovalbumen (OVA)-induced airway inflammation [10]. *Sm29* is a membrane-bound glycoprotein on the adult worm’s and lung-stage schistosomula’s tegument [11]. It has been suggested that proteins secreted by the membrane and tegument of the *S. mansoni* adult worm induce immunoregulatory responses as they are in intimate contact with host tissues [12]. Indeed, stimulation of Peripheral Blood Mononuclear Cells (PBMCs) from asthmatic patients with recombinant *Sm29* (r*Sm29*) increased the frequency of regulatory T cells and IL-10 production [13], evidencing the immunomodulatory ability of Sm29 among *S. mansoni* antigens.

As mentioned above, the cellular immune response observed in CL patients, characterized by elevated production of inflammatory cytokines and lack of immunoregulation, is a hallmark of CL [2]. Co-culture of PBMCs from CL patients with leishmania antigen plus r*Sm29*, tetraspanin 2 (SmTSP-2), and PIII, a fraction of *S. mansoni* soluble adult worm antigen (SWAP), significantly decreased the production of both IFN-γ and TNF and increased the production of IL-10 [14]. rSm29, in particular, is a potent inducer of IL-10 [15] and stimulation of monocyte-derived Dendritic Cells (DCs) from CL patients with r*Sm29* upregulated IL-10 and IL10R, corroborating earlier findings regarding the immunoregulatory properties of *S. mansoni* tegument antigens [16].

A recent clinical trial evaluated the efficacy of a topical formulation of recombinant *Sm*29 functionalized in gold nanoparticles (AuNPs-Cys-*Sm*29) combined with Sb^v^ in treating CL in Brazil [17]. CL patients were treated with systemic Sb^v^ and AuNPs-Cys-*Sm*29, the latter applied topically to lesions. The combination of AuNPs-Cys-*Sm*29 plus Sb^v^ significantly improved the clinical outcome at day 90, decreasing the healing time. Adding r*Sm29* to the standard treatment also reduced granzyme B levels [17], a hallmark of CL immunopathology [2]. We explored this combination treatment in a preclinical model of CL caused by *L. braziliensis.* We chose this combination (gold nanoparticles and r*Sm*29) due to the immunomodulatory properties of r*Sm29* as mentioned above, and because gold nanoparticles (AuNPs) are a promising platform for antigen delivery and immunomodulation. They possess intrinsic adjuvant activity, promoting dendritic cell maturation and antigen uptake, enhancing cytokine secretion, and boosting both humoral and cellular immune responses. Moreover, their properties protect antigens from enzymatic degradation, enabling controlled release at the target site, and facilitate lymphatic trafficking for efficient antigen presentation to T cells [18,19]. We show that topical AuNPs-Cys-*Sm*29 combined with systemic Sb^v^ reduced pathology and significantly decreased the parasite load *in situ* in *L. braziliensis*-infected BALB/C mice. The production of inflammatory mediators was also modulated, consolidating *S. mansoni* r*Sm29* as a novel therapeutic alternative for CL, worthy of further clinical evaluations.

## Methods

### Ethics statements

Six- to eight-week-old female BALB/c mice were obtained from the IGM-FIOCRUZ animal facility, where they were maintained under pathogen-free conditions. All animal experimentation was conducted according to the Guidelines for Animal Experimentation established by the Brazilian Council for Animal Experimentation Control (CONCEA). The local Institutional Review Board approved all the procedures involving animals for Animal Care and Experimentation (Comitê de Ética no Uso de Animais (CEUA)-IGM-FIOCRUZ-003/2017).

### Production and recovery of recombinant *Sm*29

As previously described, recombinant *Sm*29 was produced in the *E. coli* system [20]. Briefly, *E. coli* BL21 cells containing the *Sm*29 construction were cultured at 37 ºC until achieving an OD_600nm_ of 0.6-0.8. Gene expression was induced by adding isopropylthiogalactoside (IPTG) to a final concentration of 1 mM, and was carried out for 4 hours at 37 ºC at 180 rpm. Cells were harvested, treated in lysis buffer, submitted to sonication, and supernatant containing *Sm*29 was submitted to affinity chromatography on a Ni-Sepharose column (Hitrap chelating 5 mL) using an AKTA explorer chromatography system (GE Healthcare). Purified protein was dialyzed against PBS pH 7.0, and protein concentration was determined using the BCA Protein Assay Kit (Thermo Fisher Scientific), according to the manufacturer’s instructions. Purity was assessed by SDS-PAGE [21].

### Synthesis and functionalization of gold nanoparticles

As previously reported [17,22], the synthesis and functionalization of AuNPs with cysteamine (AuNPs-Cys) were performed with adjustments. Briefly, tetrachloroauric acid solution (HAuCl_4_ - Sigma) and cysteamine (Sigma) were prepared to the final concentrations of 1.5 mM and 2 mM, respectively, and homogenized and protected from light for the controlled synthesis of AuNPs-Cys. The reaction was determined by a change of color from yellow to brown with a slimy appearance. Sodium borohydride (NaBH_4_ - Sigma) was added to a final concentration of 0.34 mM, and the final solution was purple, with a non-viscous appearance, indicating that nanoparticles are well dispersed in the solution (non-aggregated). The AuNPs-Cys complex was centrifuged at 4,500 g for 25 min and washed with Milli-Q water to remove the unbound cysteamine. AuNPs-Cys were characterized by UV-VIS assessment at 400–1000 nm using Milli-Q water as a blank, and solutions were maintained at 4 °C until use.

### Functionalization of AuNPs-Cys to S*m*29 and carbopol gel preparation

AuNPs-Cys solution was previously submitted to sonication for ten minutes for total disruption of aggregates and then incubated overnight with a solution containing 550 µg/ml of recombinant *Sm*29 at room temperature under constant agitation for protein functionalization. The AuNPs-Cys-*Sm*29 complex was centrifuged at 4,500 g for 25 min and washed with Milli-Q water for unbound protein removal. The final yield of rSm29 protein was determined by ELISA, as follows. AuNPs-Cys-*Sm*29 samples were serially diluted and used to coat 96-well plates. Unbound proteins were washed out (PBS containing 0.05% Tween 20), and plates were blocked with a 10% FBS solution for 2 hours at room temperature. Then, the plates were washed and incubated with anti-His tag antibodies against the protein (1:5000) for 2 hours. Color reaction was induced by 3,3’,5,5’-Tetramethylbenzidine, and H_2_SO_4_ solution was used to stop the reaction. Plates were read at 450 nm in an ELISA plate reader (BioRad). Protein concentration was determined by interpolating detected absorbances with an *Sm*29 standard curve. Finally, AuNPs-Cys-Sm29 solutions were diluted to a final concentration of 5 and 10 µg/mL, and gels were prepared by mixing the protein complex with Carbopol 996 (1% w/v) with constant magnetic stirring for 1 h 30 min. As vehicle controls, AuNPs-Cys were complexed with Carbopol, as above, in the absence of Sm29. After obtaining a gel-like homogenous mixture, aminomethyl propanol (C_4_H_11_NO, 0.65% v/v, Sigma) was added to adjust the pH and consistency of the final gel formulation. Gels were kept at 4 ºC until use.

### Parasite culture

*Leishmania braziliensis* parasites (strain MHOM/BR/00/BA788) were grown in Schneider’s insect medium (GIBCO) supplemented with 20% heat-inactivated FBS, 2 mM glutamine, 100 U/ml penicillin, and 100 mg/ml streptomycin. Parasites were grown in airtight flasks (non-vented), incubated upright in a 25 °C incubator.

### Infection and therapeutic regimen

BALB/c mice (n = 9–10 mice per group) were infected with 10^5^ stationary-phase promastigotes in the left ear using a 27.5-gauge needle. Ear thickness was measured weekly using a digital caliper (Thomas Scientific). Mice were randomly assigned as follows: no treatment (untreated), topical treatment with AuNPs-Cys-Sm29 (5 or 10µg/ml) alone or combined with Sb^v^ (Meglumine Antimonate), 50 mg/kg/day, intraperitoneal; topical treatment with vehicle (AuNPs-Cys) alone or vehicle (AuNPs-Cys) + Sb^v^. Approximately 20ul of gel was applied (smeared onto lesions, to cover the lesion area). Mice were observed for 30 minutes to ensure the gel was not rubbed off following application. Topical treatment and systemic Sb^v^ administration were performed three times a week for four consecutive weeks. Ear thickness continued to be recorded weekly as a surrogate for lesion development. At six weeks post-infection, mice treated with vehicle (AuNPs-Cys) + Sb^v^ and mice treated with AuNPs-Cys-Sm29 + Sb^v^ were euthanized. Ears were removed postmortem and fixed in 10% formaldehyde. After 12–24 hours of fixation, tissues were processed and embedded in paraffin, and 5μm sections were stained with hematoxylin and eosin (H&E) and analyzed by light microscopy. Alternatively, mice (n = 6) were euthanized and parasite load in ears and draining lymph nodes (dLN) was determined by limiting-dilution analysis, as described previously [23].

### Cytokine quantification

Mice (n = 6) were euthanized at 6 weeks post-infection, and dLNs were homogenized individually in RPMI medium supplemented with 10% fetal bovine serum (FBS), 100 U/mL of penicillin, 100 mg/mL of streptomycin, and 2.5% HEPES (all from Invitrogen). Cell suspensions derived from each dLN (5 × 10^6^/mL) were stimulated with *L. braziliensis* soluble leishmania antigen (SLA, 12 µg/mL) for 48 hours. Control cultures were left unstimulated. Cytokine levels in culture supernatants were determined by Cytometric Bead Array (CBA) Mouse Inflammation Kit (BD Biosciences), according to the manufacturer’s instructions.

### Flow cytometry analysis

Mice (n = 6) were euthanized at 6 weeks post-infection. Treated ears were collected, separated into their ventral and dorsal layers, and incubated in RPMI medium (Gibco) with Liberase (Roche, 250 µg/ml) for 90 minutes at 37°C/ 5% CO2. Following incubation, the enzyme reaction was stopped using RPMI media containing 10% FBS. After that, the ears were homogenized in a cell strainer (40 μm, BD Pharmingen), and an aliquot of the cell suspension was used to determine parasite load. Mouse-ear cell suspensions were incubated with Cell Stimulation Cocktail (Invitrogen), Golgi Plug (BD Pharmingen), and Monensin (Invitrogen). Before surface and intracellular staining, cells were washed and stained with live/dead Fixable Viability Dye (Thermo Fisher), according to manufacturer instructions, then stained for CD45 (AF700), TCR-β (FITC), and CD4 (APC) expression (all Invitrogen). For intracellular cytokine analysis, surface-stained ear cells were fixed using Fixperm buffer, permeabilized and stained using Permwash buffer (both from Invitrogen) with specific fluorochrome-conjugated mAbs against TNF (eF450, Invitrogen), IFN-γ (PE, Invitrogen), and IL-10 (BV510, BD Biosciences). Samples were acquired on a BD Fortessa machine and analyzed using FlowJo software (TreeStar Inc., Ashland, OR). The absolute numbers of the specific cytokine-producing CD4^+^ T cells relative to the total ear leukocytes were calculated by multiplying the percentage of each gated population by the total number of viable ear cells determined by hemocytometer counts.

### Statistical analysis

The disease course was plotted individually for mice in all experimental and control groups. Comparisons between two groups were performed by Mann–Whitney (non-parametric t-test), and comparisons among more than two groups were performed by Kruskal–Wallis. Disease burden was calculated as the Area Under the Curve showing ear thickness, obtained for each mouse. Data are presented as mean ± standard deviation. Analyses were conducted using Prism (GraphPad, V 5.0), and a p-value ≤ 0.05 was considered significant.

## Results

### Production of a carbopol gel containing r*Sm*29 functionalized with gold nanoparticles

**Fig 1:**
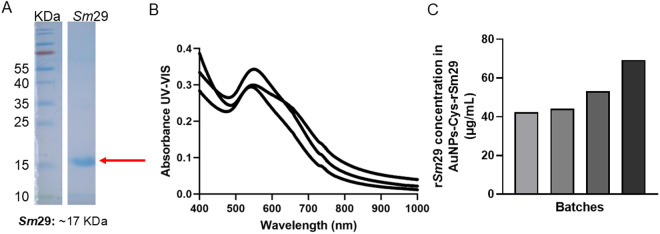
Production of recombinant Sm29 protein from Schistosoma mansoni functionalized with gold nanoparticles. (A) Recombinant Sm29 was expressed in a heterologous system and purified by affinity chromatography. Purity was assessed by SDS-PAGE 15% and Coomassie blue staining. The red arrow indicates 1 µg of pure Sm29 with approximately 17 KDa, as expected. KDa represents the molecular ladder. (B) UV-VIS assessed gold nanoparticles functionalized with cysteamine (AuNPs-Cys) at 400-1000 nm, and three representative spectra are displayed. (C) The AuNP-Cys-Sm29 complex concentration was determined by ELISA using anti-His tag antibodies against the protein (1:5000), and the absorbance detected was interpolated with a Sm29 standard curve. Four representative results of each batch yield are displayed in µg/mL. For the experiments, gel concentrations were adjusted to 5 or 10 µg/mL before use.

r*Sm*29 protein from *Schistosoma mansoni* was produced by heterologous expression in an *Escherichia coli* system, and affinity chromatography was performed for protein recovery. The purified protein was assessed by SDS-PAGE (Fig 1A), and an elevated degree of purity was observed as only a single band, at approximately 17 KDa, was detected. Gold nanoparticles were produced and functionalized with a cysteamine linker as described in the methods section, and the AuNPs-Cys complex was assessed by UV-VIS at 400–1000 nm for spectra characterization (Fig 1B). Spectra analyses of three representative batches demonstrated maximum absorbance at approximately 541 nm, characteristic of spherical nanoparticles. The lack of prominent peaks and non-specific peaks indicated homogeneity of size distribution. The formation of a slight shoulder at 588–646 nm in the spectra indicates cysteamine functionalization. Later, r*Sm*29 was functionalized into AuNPs-Cys (AuNPs-Cys-*Sm*29), and an ELISA assay was used to determine the final functionalized protein concentration (Fig 1C). Four representative batches were analyzed, and the bars demonstrate the different yields detected (40–70 µg/mL). As described in the methods, AuNPs-Cys-*Sm*29 bioconjugate concentration was adjusted to the final Carbopol gel formulation.

### The therapeutic effect of topical Sm29 in experimental CL

**Fig 2:**
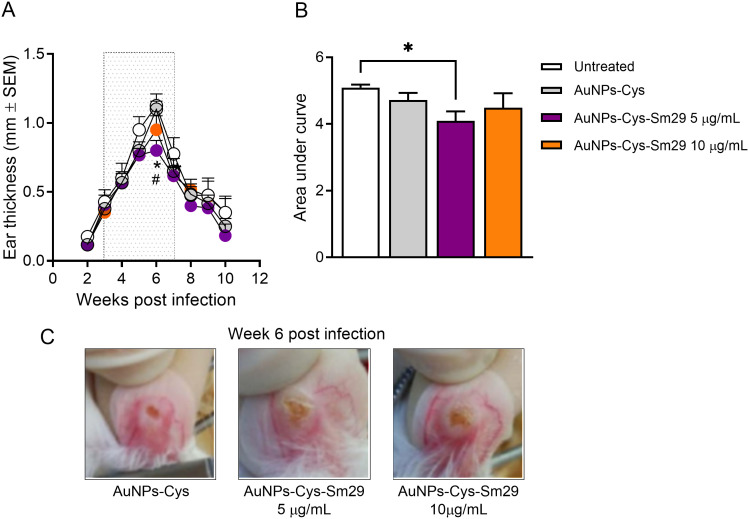
Topical AuNP-Cys-Sm29 reduces infection in mice infected with *L. braziliensis.* Mice (n = 6) were infected with *L. braziliensis* in the ear dermis, and three weeks later, mice were treated with topical AuNP-Cys-Sm29 (at 5 or 10μg/ml) for four weeks (boxed area). Control mice were left untreated (n = 4) or were treated with vehicle (AuNP-Cys, n = 6), and lesion development was measured weekly. Data are from a representative experiment performed with six mice per group. *p = 0.0381, AuNPs-Cys-Sm29 (5 µg/mL) vs Untreated. #*p* = 0.0216, AuNPs-Cys-Sm29 (5 µg/mL) vs AuNPs-Cys. (B) Area under curves (AUC) shown in (A). *p = 0.0469, AuNPs-Cys-Sm29 (5 µg/mL) vs Untreated. (C) Pictures depict lesions at 6 weeks post-infection. Data are from a representative experiment.

Mice were inoculated in the ear dermis with stationary-phase promastigotes of *L. braziliensis*, and three weeks later, the AuNPs-Cys-*Sm*29 was topically applied to lesions three times a week at 5 and 10 µg/mL. A thin layer of the AuNPs-Cys-*Sm*29 gel was applied throughout the ventral surface of the ear, covering the ulcerated lesion. Treatment with AuNPs-Cys-*Sm*29 at 10 µg/mL did not significantly reduce ear thickness, unlike the AuNPs-Cys-*Sm*29 at 5 µg/mL preparation. Using this lower concentration, we observed a significant decrease in ear thickness three weeks after the onset of treatment (six weeks post-infection) (Fig 2A), compared to untreated mice and mice treated with AuNPs-Cys alone. The disease burden of mice treated with AuNPs-Cys-*Sm*29 at 5 µg/mL, calculated by the area under the curve (AUC), was significantly reduced compared to untreated controls (Fig 2B). At six weeks post-infection, dermal lesions were visibly larger in mice treated with AuNPs-Cys (Fig 2C). Still, seven weeks post-infection, lesions began to self-heal in all groups, as previously described [23]. Additionally, since the topical application of the vehicle (AuNP-Cys) alone did not modify the course of the disease (Fig 2A), in subsequent experiments, the control group consisted of mice treated with the vehicle only.

### Topical Sm29, in combination with systemic Sb^v^, curbs lesion development and modulates the inflammatory response

**Fig 3:**
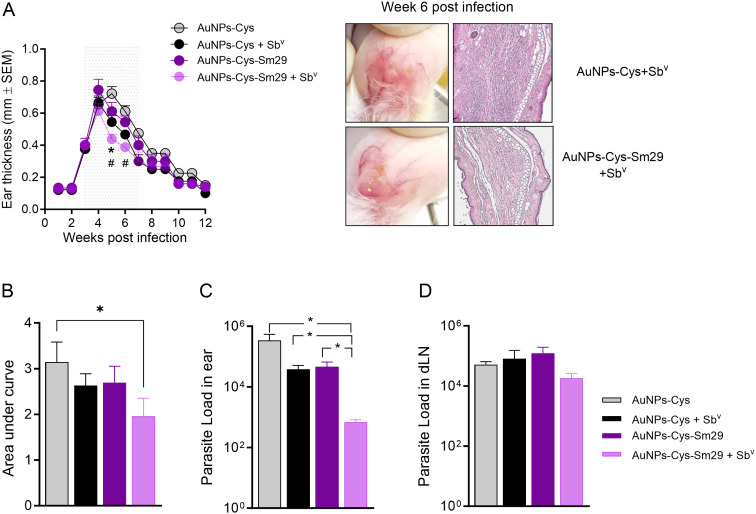
Combination treatment of topical AuNP-Cys-Sm29 + Sb^v^ reduces lesion development and parasite load *in vivo.* Mice (n = 9–10) were infected with *L. braziliensis* in the ear dermis, and three weeks later, mice were treated with topical AuNP-Cys-Sm29 (5μg/ml) alone or in combination with Sb^v^ (50 mg/kg/day) for four weeks (boxed area). Control mice (n = 9–10) were treated with vehicle (AuNP-Cys) alone or in combination with Sb^v^. (A) The course of lesion development was measured weekly. *p = 0.0281, AuNP-Cys-Sm29 + Sb^v^ *vs.* AuNP-Cys + Sb^v^ (week 5 post infection); #*p* = 0.0002, AuNP-Cys-Sm29 + Sb^v^ *vs*. vehicle (AuNP-Cys) alone (weeks 5 and 6 post infection). Six weeks post-infection, mice (n = 3–4) treated with AuNP-Cys-Sm29 + Sb^v^ or with AuNP-Cys + Sb^v^ were euthanized, and ears were stained with H&E and analyzed by optical microscopy under 20X magnification. Representative sections are shown. (B) Remaining mice were followed up until week 12 for the measurement of ear thickness and calculation of the disease burden (AUC). *p = 0.0299; AuNPs-Cys-Sm29 + Sb^v^ vs. vehicle (AuNP-Cys) alone. Alternatively, six weeks post infection, mice were euthanized (n = 6) and parasite load was determined at the inoculation site (C) and the dLN (D), 6 weeks post-infection, by limiting dilution analysis. *p = 0.0249; AuNP-Cys-Sm29 + Sb^v^ *vs.* AuNP-Cys alone; *p = 0.0167, AuNPs-Cys-Sm29 + Sb^v^ *vs.* AuNP-Cys + Sb^v^; *p = 0.0249, AuNPs-Cys-Sm29 + Sb^v^ *vs.* AuNP-Cys + Sm29. Data are from a representative experiment.

We next investigated whether a combination treatment consisting of topical AuNPs-Cys-*Sm*29 at 5 µg/mL + Sb^v^ (50mg/kg) would improve disease outcomes in mice infected with *L. braziliensis*. Three weeks following parasite inoculation, AuNPs-Cys-*Sm*29 was topically applied to dermal lesions, as described, and Sb^v^ was administered in parallel, intraperitoneally, three times a week for four weeks. The combination treatment AuNPs-Cys-*Sm*29 + Sb^v^ significantly (p < 0.05) reduced ear thickness (five weeks post-infection) compared to mice treated with vehicle (AuNP-Cys) + Sb^v^ (Fig 3A). Overall, we observed a milder inflammatory infiltrate in lesions of mice treated with AuNPs-Cys-*Sm*29 + Sb^v^ compared to the ears of mice treated with AuNPs-Cys + Sb^v^ (Fig 3A). Treatment with AuNPs-Cys-*Sm*29 + Sb^v^ significantly reduced the disease burden compared to mice treated with AuNPs-Cys (Fig 3B). On the other hand, disease burden was not significantly different comparing the combination treatment and individual variables (AuNPs-Cys + Sb^v^ or AuNPs-Cys-Sm29) (Fig 3B). Combination treatment of AuNPs-Cys-*Sm*29 + Sb^v^ significantly (p < 0.05) reduced the parasite load at the inoculation site compared to all other groups (Fig 3C). Parasite load in draining lymph nodes was similar in all groups, irrespective of the treatment strategy (Fig 3D). Thus, combination therapy consisting of AuNPs-Cys-*Sm*29 + Sb^v^ reduces CL lesion development *in vivo*, accompanied by a significant decrease in parasite load at the injection site.

**Fig 4:**
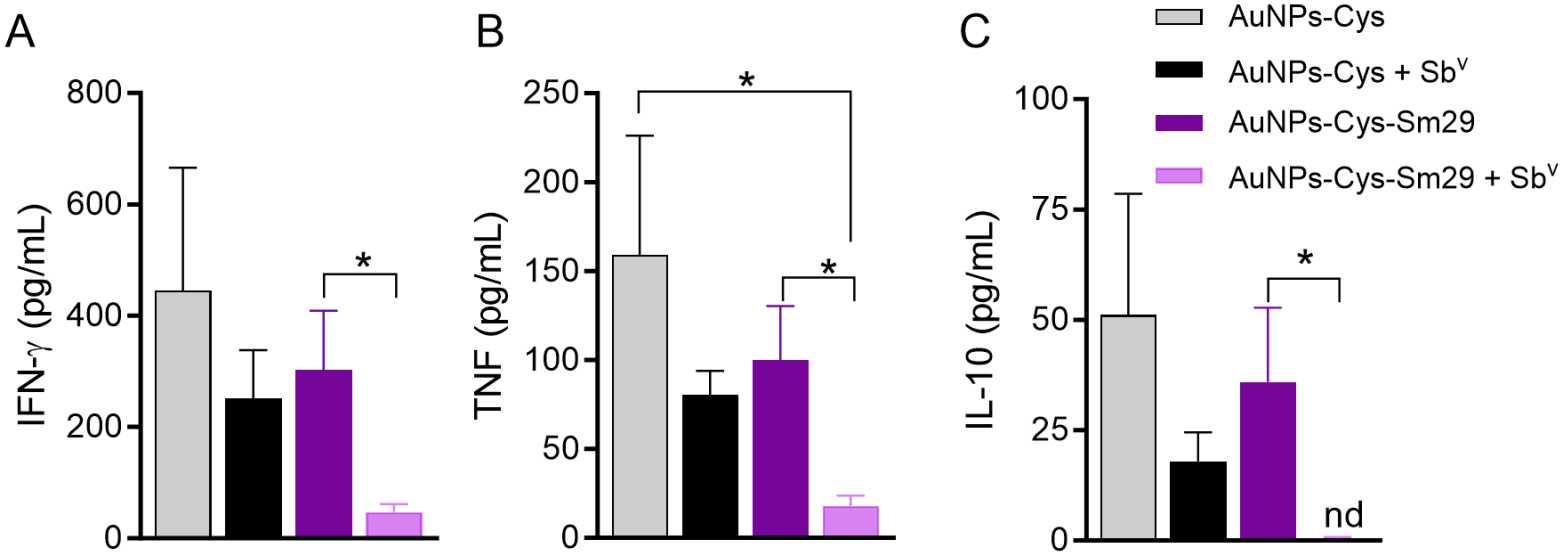
Combination treatment of topical AuNP-Cys-Sm29 + Sb^v^ modulates cytokine production. Mice (n = 6) were infected with *L. braziliensis* in the ear dermis, and three weeks later, mice were treated with topical AuNP-Cys-Sm29 (5μg/ml) alone or in combination with + Sb^v^ (50 mg/kg/day) for four weeks. Control mice (n = 6) were treated with vehicle (AuNP-Cys) alone or in combination with Sb^v^. Mice were euthanized six weeks after parasite inoculation, and cells from draining lymph nodes were restimulated in vitro. Levels of cytokines were determined in culture supernatants. (A) IFN-γ: *p = 0.0317, AuNPs-Cys-Sm29 + Sb^v^ *vs.* AuNP-Cys + Sm29; (B) TNF: *p = 0.0159, AuNPs-Cys-Sm29 + Sb^v^ *vs.* AuNP-Cys + Sm29; *p = 0.0159, AuNPs-Cys-Sm29 + Sb^v^ *vs.* AuNP-Cys, and (C) IL-10, *p = 0.0476; AuNPs-Cys-Sm29 + Sb^v^ *vs.* AuNP-Cys + Sm29. Data are from a representative experiment.

**Fig 5:**
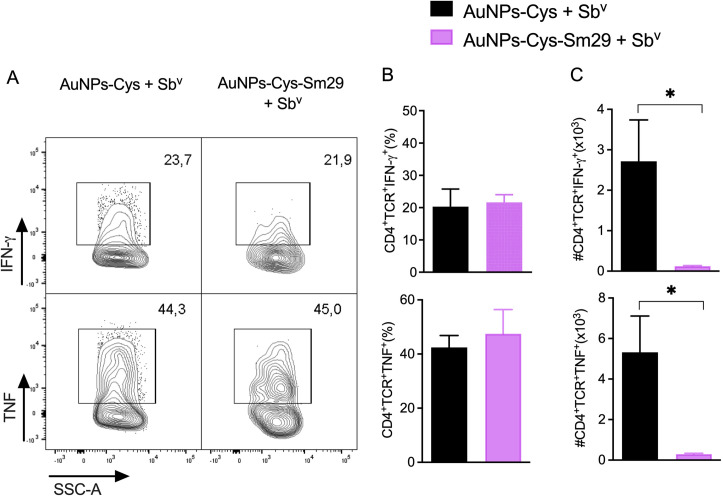
Frequency of cytokine-producing CD4 + T cells in mice treated with topical AuNP-Cys-Sm29 + Sb^v^. Mice (n = 6) were infected with *L. braziliensis* in the ear dermis, and three weeks later, mice were treated with topical AuNP-Cys-Sm29 (5μg/ml) + Sb^v^ (50 mg/kg/day) for four weeks. Control mice (n = 6) were treated with vehicle (AuNP-Cys) + Sb^v^. Mice were euthanized six weeks after parasite inoculation, and cells from the lesion site (ear) were stained for IFN-γ or TNF. (A) Representative contour plots, frequency of CD4^+^ T cells expressing IFN-γ or TNF. (B) Percentage of CD4^+^IFN-γ^+^ or CD4^+^TNF^+^ cells in mice treated with AuNP-Cys-Sm29 + Sb^v^ or AuNP-Cys + Sb^v^. (C) Number of CD4^+^IFN-γ^+^ or CD4^+^TNF^+^ cells in mice treated with AuNP-Cys-Sm29 + Sb^v^ or vehicle (AuNP-Cys) + Sb^v^; *p = 0.0286. Cell populations were pregated on Singlets/Live/CD45^+^/TCRb^+^/CD4^+^. Data are from a representative experiment.

Following the observation that AuNPs-Cys-*Sm*29 + Sb^v^ ameliorated lesion development and reduced parasite load at the injection site (Fig 3), we evaluated the cellular immune response in treated mice. Mice were euthanized three weeks after treatment initiation, the time point at which we saw significant differences comparing groups treated with AuNP-Cys-Sm29 and AuNP-Cys (Fig 3), dLN cells were restimulated, and cytokine levels were determined in culture supernatants. The addition of AuNPs-Cys-*Sm*29 to systemic Sb^v^ use significantly reduced levels of IFN-γ (Fig 4A), TNF (Fig 4B), and IL-10 (Fig 4C) compared to mice treated with AuNPs-Cys-*Sm*29 only. Since the parasite load was significantly reduced at the lesion site (Fig 3C) in mice treated with AuNPs-Cys-*Sm*29 + Sb^v^, we evaluated the frequency of cytokine-secreting CD4^+^ T cells at the lesion site (ear). Similar frequencies of CD4^+^IFN-γ^+^ and CD4^+^TNF^+^ T cells were observed in mice treated with AuNPs-Cys-*Sm*29 + Sb^v^ and in mice treated with vehicle (AuNPs-Cys) + Sb^v^ (Fig 5A). However, the absolute number of IFN-γ and TNF-producing CD4^+^ T cells decreased by 21.7-fold and 18.1-fold, respectively, upon the combination use of AuNPs-Cys-*Sm*29 + Sb^v^, in comparison to mice treated with AuNPs-Cys + Sb^v^ (Fig 5C). These results show that topical AuNPs-Cys-*Sm*29 application + systemic Sb^v^ inhibits lesion development and decreases the parasite load, paralleled by reduced IFN-γ and TNF-secreting cells.

## Discussion

In CL, ulcer development results from the combined presence of Th1 cytokines (such as IFN-γ and TNF), IL-1β, and cytotoxic activity from CD8 + T cells. The ability of *Leishmania* parasites to evade these cytotoxic mechanisms allows for the parasite and antigen persistence, eliciting a pronounced inflammatory response and tissue degradation [2]. Research has shown that host-directed therapies and chemotherapy induce favorable outcomes in CL patients [24–27]. This study explored a combination treatment consisting of topically applied Sm29, an immune modulator extracted from *S. mansoni*, plus Sb^v^ in a preclinical model of CL caused by *L. braziliensis*.

Mice infected with *L. braziliensis* and treated with topical AuNPs-Cys-*Sm*29 showed a significant decrease in ear thickness (a proxy for active local inflammation) three weeks after the onset of treatment. Later, when we probed a combination treatment of topical AuNPs-Cys-*Sm*29 plus Sb^v^, we observed a significantly reduced ear thickness accompanied by a milder inflammatory infiltrate at the lesion site. The combination treatment also significantly decreased the parasite load at the injection site but not in draining lymph nodes. While we have not, in the present study, evaluated the effect of treatment with Sb^v^ alone, evidence from the clinical trial conducted with AuNPs-Cys-*Sm*29 shows an additive effect compared to standard therapy with Sb^v^ alone. In this trial, the cure rate was 71% in subjects treated with AuNPs-Cys-Sm29 plus Sb^v^ and 43% in patients treated with Sb^v^ plus placebo or Sb^v^ alone [17].

Herein, the choice of Sm29 formulation in AuNPs-Cys was based on the capability of nanoparticles to deliver different types of biomolecules, including recombinant proteins, drugs, and nucleotides [28], plus the possibility of nanoparticle customization for the delivery of biomolecules [29]. The choice of gold as an inert material and the route of application (topical) aimed at reducing possible toxicity issues. Moreover, our previous work has shown that Sm29 is not cytotoxic, even when applied systemically [30–32]. The combination treatment consisting of topical AuNPs-Cys-*Sm*29 + Sb^v^ reduced the production of mediators such as IFN-γ and TNF compared to mice treated with AuNPs-Cys-*Sm*29 alone. Thus, in experimental CL, the combination of AuNPs-Cys-*Sm*29 + Sb^v^ inhibited lesion development and decreased the parasite load without increasing the production of IFN-γ or TNF (in dLNs). In the present study, we have not measured Th-2-related cytokines; thus, we do not know whether the Sm29 application enhanced IL-4 production. For example, IL-10 levels were also low in mice treated with AuNPs-Cys-*Sm*29 + Sb^v^. We can speculate that Sb^v^ contributed to parasite killing, and the resulting decrease in antigens also curbed the cellular response. Indeed, in mice treated with AuNPs-Cys-*Sm*29 only, levels of IFN-γ, TNF, and IL-10 are significantly higher compared to AuNPs-Cys-*Sm*29 + Sb^v.^ Additionally, AuNPs exhibit antioxidant activity, inducing more pronounced effects in a model of acute kidney injury compared to N-acetylcysteine [33]. Thus, we cannot exclude that the anti-inflammatory effect observed in the present study also results from the presence of AuNPs in parallel to Sm29.

It is well established that in CL caused by *L. braziliensis,* CD8^+^ T cells cause immunopathology [34,35]. Thus, studies have addressed ways to counteract CD8^+^ T cell effector functions and downstream events without impacting general T cell activation, IFN-γ production, and parasite killing. When CL lesion tissue was cultured in the presence of Glyburide, an NLRP3 inhibitor, the production of IL-1β, IL-17, and TNF was significantly reduced without altering IFN-γ production [36]. In experimental CL, topical application of Tofacitinib, which inhibits Granzyme release by CD8^+^ T cells, inhibited lesion development without changing the parasite load [7]. In a recent clinical trial conducted in CL patients, a combination treatment consisting of topical AuNPs-Cys-*Sm*29 plus intravenous Sb^v^ increased the cure rate and decreased the healing time significantly compared to Sb^v^ alone [17]. This trial evidenced the ability of topical AuNPs-Cys-*Sm*29 to modulate the overt inflammatory response observed in CL lesions, highlighting the potential use of immune modulators to treat this condition. In this trial, skin biopsies were obtained 7 days after the start of treatment. Tissue was cultured, and supernatants were used for cytokine quantification. Authors observed increased levels of IFN-γ and decreased levels of Granzyme B while TNF levels remained unaltered [17]. In our work, cytokine production by dLN cells was evaluated three weeks after initiation of treatment. Thus, we can speculate that the differences observed in our study (lower levels of IFN-γ and TNF) may reflect a later stage when the inflammatory response was lower due to the immune modulation caused by the presence of Sm29.

### Additionall

Our work showed that combining topical AuNPs-Cys-*Sm*29 + Sb^v^ modulated the inflammatory response and decreased ear thickness and the parasite load in experimental CL caused by *L. braziliensis*. However, it is important to highlight some limitations of this study, such as the precise mass of Sm29 protein administered in topical treatments and a more comprehensive evaluation of the local effects of AuNPs-Cys-Sm29 *in vivo* (absorption rate, local anti-inflammatory effects, and possible anti-oxidative effects). Also, we have evaluated two formulations (5 and 10 µg/mL of r*Sm*29) and reported only the lower concentration presented effectiveness. Our data currently do not allow mechanistic explanations underlying the different outcomes (e.g., non-linear dose-response profile, concentration-dependent cellular uptake and interactions, etc.). Thus, further dose-response studies and formulation optimizations are required to better understand the matter, including whether a higher concentration of Sm29 might favor parasite survival, given its immunomodulatory effect. Nonetheless, we provide confirmatory evidence that the functionalization of Sm29 into gold nanoparticles can be further explored for the delivery of host-directed therapy for treating CL in conjunction with chemotherapy.

## Supporting information

S1 Material The Excel file contains supplementary materials associated with the manuscript mentioned above. The information is organized across tabs, containing Figs 2–5 data.
(XLSX)
